# Supplementary material for: Social and mental health impact of COVID-19 pandemic among health professionals of Gandaki Province, Nepal: A mixed method study
Source: PLoS One. 2023 Apr 6;18(4):e0283948. doi: 10.1371/journal.pone.0283948 (PMC10079026; doi:10.1371/journal.pone.0283948)
Supplement: S1 File — (DOCX) [file pone.0283948.s001.docx]

**Social and Mental Health Impact of COVID-19 Pandemic among**

**Healthcare Professionals, Gandaki Province, Nepal**

You are invited to participate in a web-based research study on “Social and Mental Health Impact of COVID-19 Pandemic among Healthcare Professionals working in Gandaki Province of Nepal”. This study aims to explore the Social Impact of the ongoing COVID-19 Pandemic among Healthcare professionals. All healthcare professionals currently working at different public and private institutions of Gandaki province are requested to participate in this study. Participation in this study is voluntary and the participant can withdraw at any time while completing the provided Google Form. However, as this study is of a current social issue we request all the participants to provide fair and complete information as much as possible. Based on your response, we hope to reflect on the current social impact and hardship that healthcare professionals face during this crisis. The interview also includes questions about job satisfaction, fear scale for COVID-19, and PHQ-9 to screen depressive symptoms among the health professionals. All the information that you provide will be kept confidential and only the researchers involved will have access to it for a limited period. The information provided will only be used for the study purpose and will be destroyed after the completion of this study. We hope you will participate in this study since your information is very important to us. Participating in this study may not benefit you directly, but it will help to learn the current Social and Mental Health Impact of the COVID-19 Pandemic among Healthcare professionals.

If you agree to participate in this study and would like to fill the questionnaires, please provide your email as your approval for this study.______________________________

**Part 1: Background**

1. Gender:
2. Male b. Female c. Trans gender d. . Prefer not to say
3. Age (In completed years ) ​____________________
4. Home Address/permanent address ___________________________
5. Temporary Address (Currently Living/working At?) ____________________
6. What is your marital status?
   1. Unmarried
   2. Married
   3. Separated
   4. Divorced
   5. Widow
7. What is your highest academic quantification?
   1. TSLC (E.g: AMN, CMA...)
   2. Diploma (E.g: H.A, Staff Nurse ...)
   3. Bachelor (E.g: MBBS, BDS, B.Pharm, BSC Nursing, BMLT, BPH )
   4. Masters and above (E.g: MD, MPharm, MSC Nursing, MLT, MPH )
8. What is your job position? ?(E.g: ANM, HA, Lab Assistants, Nurse, Pharmacist, Medical Officer, Consultant) _____________________
9. Years of Service contributed in the Health sector _____________________
10. Is your current job categorized in Government/Public Service or Private practice
    1. Government/public service / in gov. organization
    2. Private practice/organization
11. Type of service
    1. Permanent
    2. Temporary
    3. Contract
    4. Own Business (example: Medical store, Own clinics)
    5. Volunteer
    6. Other:_________________
12. There is appropriate Insurance and security system in your organization for you?
    1. Yes  ​​​​
    2. No
13. Do your workplace adopt adequate preventive measures against COVID-19 (Such as the use of masks, physical distancing, and frequent hand-wash/ hand sanitizer facilities)
    1. Yes  ​​​​
    2. No
14. Do you think COVID-19 have impacted your family relationship? (either in a positive or negative way )
    1. Yes  ​​​​
    2. No
15. If yes, what is the impact . (multiple choice question)

|  |  | 1. Yes | 1. No |
| --- | --- | --- | --- |
| 14.1 | Feeling distanced by the family members |  |  |
| 14.2 | Feeling worried that member of the family will get infected |  |  |
| 14.3 | Feeling more close and respected by the family members |  |  |

1. Do you think COVID-19 have impacted your social relationship with friends and relatives? (either in a positive or negative way)  ​​​​
   1. Yes
   2. No
2. If yes, what is the impact (multiple choice question)

|  |  | 1. Yes | 0. .No |
| --- | --- | --- | --- |
| 16.1 | Sense of being distanced by a friends and relatives due to your profession as HW |  |  |
| 16.2 | Sense of being discriminated by a friend and relatives due to your profession as HW |  |  |
| 16.3 | Sense of being respected by friends and relatives due to your profession |  |  |
| 16.4 | Sense of fear while being close to them |  |  |

1. Do your think COVID-19 have impacted your social relationship with community people (either in a positive or negative way)
   1. Yes
   2. No
2. If yes, what is the impact (multiple choice question)

|  |  | 1. Yes | 0. .No |
| --- | --- | --- | --- |
| 18.1 | Sense of being distanced by a community people or society due to your profession as HW |  |  |
| 18.2 | Sense of being discriminated by a community people or society due to your profession as HW |  |  |
| 18.3 | Sense of being respected by community people or society due to your profession |  |  |
| 18.4 | Sense of while being close to community people |  |  |

1. Do you live in rent or own a house
   1. Rent
   2. Own House
   3. Hostel
   4. Quarters
   5. Other
2. If not in your, own house, Do your feel COVID-19 have impacted your social relationship with house owner (either in a positive or negative way)?

|  |  | 1. Yes | 0. .No |
| --- | --- | --- | --- |
| 20.1 | Sense of being distanced by house owner due to your profession as HW |  |  |
| 20.2 | Sense of being discriminated by the house owner due to your profession as HW |  |  |
| 20.3 | Sense of being respected by the house owner due to your profession |  |  |
| 20.4 | Sense of fear while getting close to them |  |  |

1. Have you recently been discriminated or badly treaded by anyone due to your profession of being health worker during this COVID-19 pandemic
   1. Yes
   2. No
2. If yes. By whom (Multiple Choice)

|  |  | 1. Yes | 0. .No |
| --- | --- | --- | --- |
| 22.1 | Family and relatives |  |  |
| 22.2 | Friend and colleagues |  |  |
| 22.3 | Community People and Neighbors |  |  |
| 22.4 | Community leaders |  |  |

1. What might be the reason for them to treat you that way? (Multiple Choice)

|  |  | 1. Yes | 0. .No |
| --- | --- | --- | --- |
| 23.1 | Have no idea why they treated you badly or differently |  |  |
| 23.2 | They fear being close to you as they feel it increase their risk of contracting COVID-19 infection |  |  |
| 2.34 | People feel HW are not playing their role properly to handle the infection (Or people are dissatisfied with Health workers) |  |  |

1. Do your think Nepal Government is providing enough resources to the HW
   1. Yes ​​​​
   2. No
2. If no, What is lacking? (Multiple Choices)

|  |  | 1. Yes | 0. .No |
| --- | --- | --- | --- |
|  | Financial support and intensives to the HW |  |  |
|  | Medical and logistic supply for COVID-19 test and symptomatic treatment |  |  |
|  | Proper PPE |  |  |
|  | Low number of health institution providing COVID-19 services |  |  |
|  | High cost for PCR test and poor community access |  |  |
|  | Proper tracking mechanism |  |  |
|  | Inadequate skilled human resources |  |  |

1. Choose your level of satisfaction with the following statements in the job.

| S.N | Statements | Level of satisfaction | | | | |
| --- | --- | --- | --- | --- | --- | --- |
|  |  | Fully  dissatisfied | Partly dissatisfied | Neutral | Partly satisfied | Fully satisfied |
| 1 | Salary/Incentives is good for my work |  |  |  |  |  |
| 2 | Pension & security Services is good |  |  |  |  |  |
| 3 | Work environment is safe |  |  |  |  |  |
| 4 | Adequacy of technical & medical equipment |  |  |  |  |  |
| 5 | Solidarity & relationship with co-workers |  |  |  |  |  |
| 6 | The way my boss handles his staff |  |  |  |  |  |
| 7 | Opportunity for trainings and education |  |  |  |  |  |
| 8 | Opportunity for promotion and progress |  |  |  |  |  |
| 9 | Opportunity to utilize skills and talents |  |  |  |  |  |
| 10 | Freedom to use my own judgments |  |  |  |  |  |
| 11 | Rest and refreshment that  I get |  |  |  |  |  |
| 12 | Recognition and reward  for good jobs |  |  |  |  |  |

 27. COVI-19 Fear Scale

| S.N |  | Strongly Disagree | Disagree | Neutral | Agree | Strongly Agree |
| --- | --- | --- | --- | --- | --- | --- |
| 1. . | I am most afraid of coronavirus-19. |  |  |  |  |  |
|  | It makes me uncomfortable to think about coronavirus-19. |  |  |  |  |  |
|  | My hands become clammy when I think about coronavirus-19. |  |  |  |  |  |
|  | I am afraid of losing my life because of coronavirus-19. |  |  |  |  |  |
|  | When watching news and stories about coronavirus-19 on social media, I become nervous or anxious. |  |  |  |  |  |
|  | I cannot sleep because I’m worrying about getting coronavirus-19. |  |  |  |  |  |
|  | My heart races or palpitates when I think about getting coronavirus-19. |  |  |  |  |  |

1. PHQ-9: Over last 2 weeks, how often have you been bothered by following problems?

|  |  | Not at all | Several Days | More than half days of week | Nearly every day |
| --- | --- | --- | --- | --- | --- |
|  | Little interest or pleasure in doing things |  |  |  |  |
|  | Feeling down, depressed or hopeless |  |  |  |  |
|  | Trouble falling or staying asleep or sleeping too much |  |  |  |  |
|  | Feeling tired or having little energy |  |  |  |  |
|  | Poor appetite or overeating |  |  |  |  |
|  | Feeling bad about yourself- or that you are a failure or have let yourself or your family down |  |  |  |  |
|  | Trouble concentrating on things as reading newspaper or watching television |  |  |  |  |
|  | Moving or speaking so slowly that other people could have noticed? Or the opposite - being so fidgety or restless that you have been moving around a lot more than usual? |  |  |  |  |
|  | Thoughts that you would be better off dead, or of hurting yourself in some way? |  |  |  |  |
