# Supplementary material for: Social and mental health impact of COVID-19 pandemic among health professionals of Gandaki Province, Nepal: A mixed method study
Source: PLoS One. 2023 Apr 6;18(4):e0283948. doi: 10.1371/journal.pone.0283948 (PMC10079026; doi:10.1371/journal.pone.0283948)
Supplement: S2 File — (DOCX) [file pone.0283948.s002.docx]

**Social and Mental health impact of COVID-19 Pandemic among Health Professionals of Gandaki Province, Nepal: A mixed method study**

**Interview Guideline for Health Professionals**

Respondent name:

Place of interview:

Date and time of interview

Name of interviewer:

**Objectives of the interview**

- To assess the social impact of COVID-19 experienced by the Healthcare professional in their family and community relationship
- To assess the expectation of the Healthcare professional from Health system and GON and level of motivation and satisfaction among Healthcare professionals during this pandemic

| **Section** | **Question** | **Remarks/things to remember for probes** |
| --- | --- | --- |
| Socio-demography | Age:  Sex:  Place of residence:  Education level:  Years of experience in the Health sector:  Current Origination:  Designation:  Year Experience in the given position: |  |
| Role in COVID-19 | 1. What are the roles that you have been engaged in after this COVID-19 pandemic? 2. What are the practices you have been adopting to prevent yourself and your family from infection? 3. Have you ever tested or considered to test for RDT or Rt-PCR? 4. Is the currently available test effective and sufficient for Nepal? | If no, why not?  Q4. If yes, how is the test process? Is it easily assessable to the public? |
| Social and mental health aspect in COVID-19 | 1. Have you experienced any changes in your social relationship with your family during this COVID-19 Pandemic? 2. Have you experienced any changes in your social relationship with your communities and friends during this COVID-19 Pandemic? 3. Health care professionals have been reported to be stigmatized and discriminated in many parts of Nepal at this time of infection, What your opinion on this? 4. Have you feeling depressed or mental stressed lately due to the pandemic? 5. What can be the role that community, leaders, and Government can play to support health workers? | Why? What changes? How did you cope with it? How are other health professionals copying with it?  What changes and why?  How did you cope with it? How are other health professionals copying with it? |
| Expectation | 1. Do you have expectations from GON in terms of social, economic, resources, or other supports at this time of Pandemic? | Local Government, Provincial Government and Federal Government |
| Satisfaction & Motivation | 1. Are you satisfied with the incentives and resources provided by the GON to tackle COVID-19 in your area? 2. What should the GON do to improve the level of satisfaction among healthcare providers? | If no, what are the things that dissatisfy you the most?  The question is for all three tires of Government of Nepal. |
| Any suggestion | To your current healthcare institution & To local government & To MOHP and GON |  |
